# Impact of Drp1-Mediated Mitochondrial Dynamics on T Cell Immune Modulation

**DOI:** 10.3389/fimmu.2022.873834

**Published:** 2022-03-31

**Authors:** Jun Song, Xiaofang Yi, Ruolin Gao, Li Sun, Zhixuan Wu, Shuling Zhang, Letian Huang, Chengbo Han, Jietao Ma

**Affiliations:** Department of Oncology, Shengjing Hospital of China Medical University, Shenyang, China

**Keywords:** dynamin-related protein 1, immunotherapy, mitochondrial dynamics, T cell exhaustion, tumor microenvironment

## Abstract

In recent years, various breakthroughs have been made in tumor immunotherapy that have contributed to prolonging the survival of tumor patients. However, only a subset of patients respond to immunotherapy, which limits its use. One reason for this is that the tumor microenvironment (TME) hinders the migration and infiltration of T cells and affects their continuous functioning, resulting in an exhausted phenotype. Therefore, clarifying the mechanism by which T cells become exhausted is of significance for improving the efficacy of immunotherapy. Several recent studies have shown that mitochondrial dynamics play an important role in the immune surveillance function of T cells. Dynamin-related protein 1 (Drp1) is a key protein that mediates mitochondrial fission and maintains the mitochondrial dynamic network. Drp1 regulates various activities of T cells *in vivo* by mediating the activation of a series of pathways. In addition, abnormal mitochondrial dynamics were observed in exhausted T cells in the TME. As a potential target for immunotherapy, in this review, we describe in detail how Drp1 regulates various physiological functions of T cells and induces changes in mitochondrial dynamics in the TME, providing a theoretical basis for further research.

## Introduction

In recent years, with our increased understanding of the immune system, immunotherapy has become an effective treatment for many types of tumors, prolonging patient survival (1). Immune cells, especially T cells, play a key role in immunotherapy (1). Different metabolic forms are required to direct the effector function of T cells at different stages (2). T cells are rapidly activated into T effector cells once antigen is detected, then die or transform into T memory cells after completion of the immune response (3). Thus, cellular metabolism must be reprogramed to acquire different phenotypes and functions. For example, increased glycolysis was observed in activated T effector cells, while increased levels of fatty acid oxidation were observed in suppressive T regulatory cells (Treg) (2). Interestingly, although elevated levels of fatty acid oxidation were observed in Treg (2), Treg metabolism was not dependent on fatty acid oxidation. The study by Raud *et al.* showed that there are pathways other than fatty acid oxidation that regulate Treg differentiation (4). Furthermore, recent data suggest that Myc is involved in regulating T cell activation and metabolic reprogramming, and that Myc is a regulator of mitochondrial oxidative metabolism (5). Although the mechanism of metabolic regulation of T cell subsets has not been fully elucidated, this review aims to link mitochondria with the various functions of T cells, providing new possibilities for improving anti-tumor immunotherapy.

**Figure 1:**
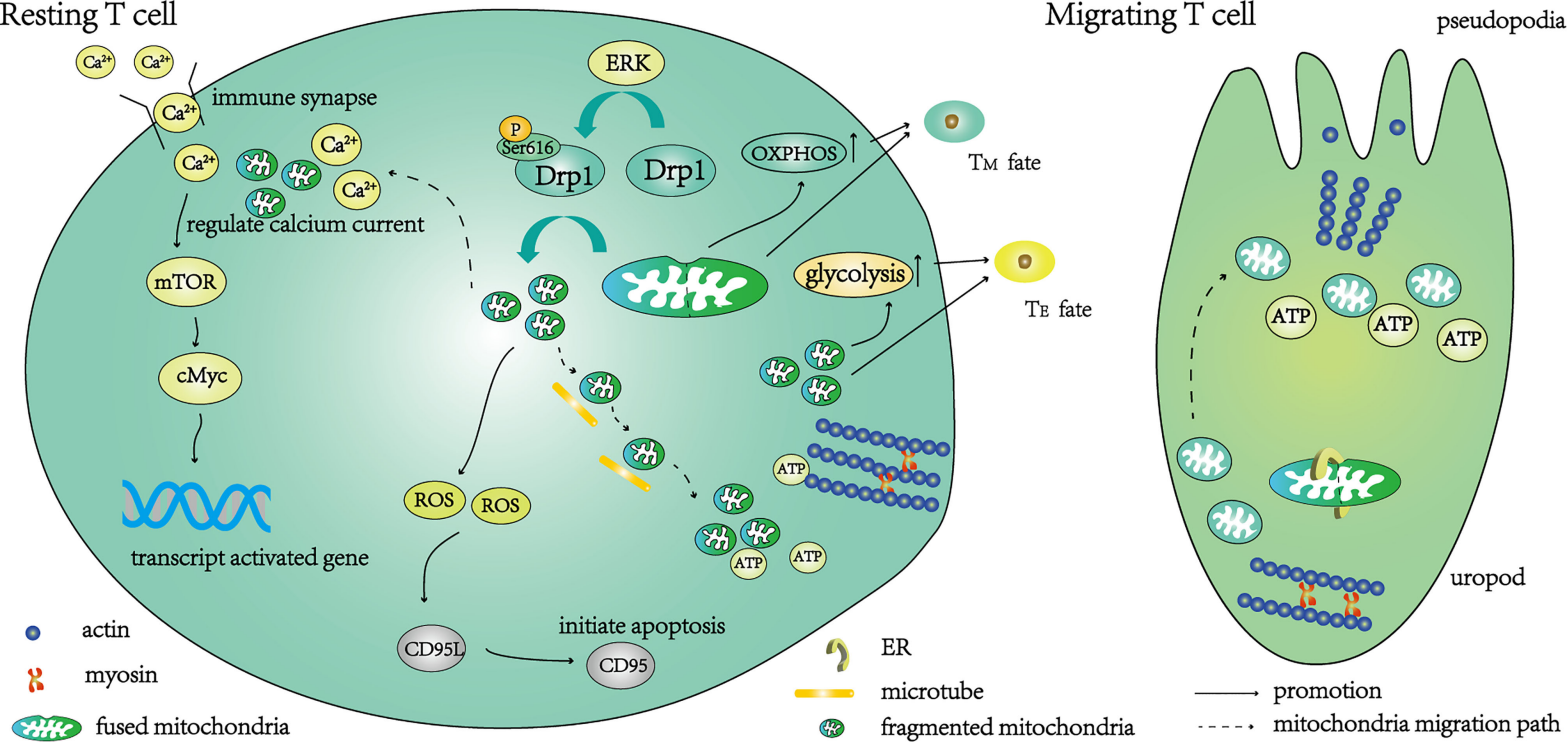
A schematic diagram of the function of T cells regulated by Drp1-mediated mitochondrial fission. Under the action of the ERK pathway, the phosphorylation of Ser616 activates Drp1, and the activated Drp1 mediates mitochondrial fission. After fragmentation, the mitochondria migrate along the microtubule to the uropod, where they provide ATP for T cell movement. The initiation of mitochondrial fission begins with contact between the endoplasmic reticulum (ER) and mitochondria, then resting T cells transition to migrating T cells. Part of the fragmented mitochondria migrates to the immune synapse, where calcium ions are absorbed to regulate calcium currents. Under a suitable calcium current, mTOR and cMyc are further activated to enhance the transcription of activation-related genes. The main metabolic pathway of these T cells is glycolysis, and the T cells differentiate into effector T cells (T_E_). Fragmented mitochondria also produce ROS, activate CD95L transcription, further activate CD95 and mediate T cell apoptosis. T cells are converted to a memory-like phenotype (T_M_) when knockdown of Drp1 inhibits mitochondrial fission and distributes fused mitochondria within T cells.

Mitochondria have a bilayer membrane structure, including an outer mitochondrial membrane (OMM) and inner mitochondrial membrane (IMM). The mitochondrial membrane comprises numerous proteins. The mitochondrial fusion proteins mitofusin (Mfn, including Mfn1 and Mfn2) (6) and optic atrophy 1 (Opa1) (7) are located in the OMM and IMM, respectively. In addition, mitochondrial anti-viral proteins (8) and mitochondrial anti-apoptotic proteins also exist in the OMM, while the electron transport chain is located on the IMM. The morphology of mitochondria is highly dynamic and the processes of fusion and fission occur continuously. Different forms of mitochondria are observed that regulate the energy metabolism of cells to adapt to the surrounding environment (9). Mitochondrial fusion is related to an increase in oxidative phosphorylation and adenosine triphosphate (ATP) production, mediated by mitochondrial fusion proteins Mfn1, Mfn2 and Opa1 (10). When needed, the fused mitochondrial network is fragmented, a process mediated by guanosine-5′-triphosphate (GTPase) and dynamin-related protein 1 (Drp1) (11). The initiation of mitochondrial fission begins with contact between the endoplasmic reticulum and mitochondria (**Figure 1**). The recruitment of Drp1 to the OMM is regulated by other proteins such as fission protein 1 (Fis1), mitochondrial fission factor (MFF), MiD49 and MiD51 (7). Among these, Fis1 is not only a receptor recruited by Drp1 from the cytoplasm to the OMM, but also promotes mitochondrial fission by negatively regulating the fusion process of mitochondria (12). Drp1 is usually located in the cytoplasm when it is inactive and is phosphorylated at serine 637 (inhibitory phosphorylation) (13). When fission is initiated, Drp1 is widely recruited to the OMM and phosphorylated at serine 616, causing mitochondrial fragmentation through the formation of oligomeric rings (14). In recent years, studies have shown that mitochondrial dynamics are crucial for regulating immune cell growth, development and migration, and the immune response (3, 15). In this review, we summarize the regulation of Drp1-mediated mitochondrial fission at different T cell stages and the changes in these physiological processes in the anti-tumor immune response, and explore the possibility of developing new therapeutic strategies targeting Drp1.

## Drp1-Mediated Mitochondrial Fission in Different T Cell Stages

### Drp1-Mediated Mitochondrial Fission and T Cell Maturation

In mice with specific Drp1 ablation during T cell development, the number of mature T cells in the thymus was reportedly decreased, whilst normal organelle function was retained and all mature T cell subsets were correctly represented (16). Therefore, knockout of Drp1 only appears to affect the number of mature T cells and has no impact on their function or differentiation into subtypes. Furthermore, the reduced proliferation of Drp1-knockout mature T cells was also observed after stimulation with antigen (16). Interestingly, this reduction in the clonal expansion rate could be reversed by activating Drp1-S616E overexpression (16). Although the underlying mechanism remains unclear, the impairment of T cell migration function may partly explain this phenomenon. T cells migrate from the cortex to the medulla in the thymus and undergo strict positive and negative selection (17). Knockout of Drp1 inhibits T cell migration, thereby leading to the accumulation of T cells in the thymic cortex (16). This, in turn, affects the screening of T cells and leads to excessive T cell clearance. It is unclear whether Drp1 is involved in regulating positive and negative selection, although knockout of Drp1 does not lead to an increase in autoreactive T cells.

### Drp1-Mediated Mitochondrial Fission and T Cell Migration

The migration of T cells depends on their unique cytoskeleton, which converts chemical signals into mechanical energy and promotes migration (18, 19). During this process, Drp1 is phosphorylated at serine 616 under the action of the mitogen-activated protein kinase pathway (14), then the mitochondria are fragmented and relocated to the uropod along the microtubules (**Figure 1**), providing ATP for the movement of myosin (20). Interestingly, the lack of Drp1 leads to the fusion of mitochondria, which is not conducive to microtubule-mediated transport, while overexpression of Drp1 can promote mitochondrial relocation and accelerate T cell migration (16). This may be due to the inability of microtubules to transport excessively large organelles, or the disturbance of mitochondrial dynamics by changes in the morphology of mitochondria (20). Impaired migration not only affects the maturation of T cells in the thymus (reducing the survival rate of thymocytes during positive selection), but also hinders the migration of T cells to the blood and secondary lymphoid organs, which play a role in immune surveillance (16).

### Drp1-Mediated Mitochondrial Fission and T Cell Activation

As a second messenger, the calcium current is indispensable for T cell activation. Mitochondria of the immune synapse ensure the calcium release-activated calcium channels remain open by absorbing calcium, keeping the calcium current at a low level most suitable for T cell activation (21, 22) (**Figure 1**). The calcium current regulates the AMP-activated protein kinase pathway (23) and the mechanistic target of rapamycin (mTOR) pathway (24) to control the metabolic reprogramming of T cells to meet the metabolic needs of the activated state (25, 26). The complete activation of Drp1 depends on calcium-dependent calcineurin-regulated serine 637 dephosphorylation (27) and activation of phosphorylated serine 616 by the mitogen-activated protein kinase pathway (16). Activated Drp1 mediates mitochondrial division and migration to immune synapses (28) (**Figure 1**). The calcium influx of Drp1-deficient T cells increases during activation, which further leads to the over-activation of AMP-activated protein kinase and a decrease in mTOR-cMyc (16). Since cMyc is necessary for transcription when metabolic genes are activated in T cells (29), its reduction may affect their metabolic transcription. In addition, the electron transport chain located in the IMM produces reactive oxygen species (ROS), which are intrinsically linked to the stability of mitochondrial dynamics, because high levels of ROS can lead to cell damage (30, 31).

### Drp1-Mediated Mitochondrial Fission and T Cell Differentiation

After activation, T cells differentiate into i) effector T cells (T_E_), which participate in immune reactions to foreign antigens, and ii) memory T cells (T_M_), which differentiate rapidly into T_E_ to participate in the immune response on subsequent exposure to the same antigen. The metabolic patterns of different T cell subsets differ greatly (32, 33). Mitochondria, as metabolic centers, affect the differentiation of T cells. During the differentiation of naïve T cells into T_E_, the metabolic mode changes from oxidative phosphorylation and fatty acid oxidation to glycolysis (34). This process depends on the precise regulation of the calcium current at immune synapses by Drp1, which promotes the transcription of glycolysis genes by maintaining the activation of mTOR/cMyc (16) (**Figure 1**). Fragmented mitochondria were predominantly observed in T_E_, and this phenotype depended on the phosphorylation of Drp1 at Ser616 to mediate mitochondrial division (35). Knockout of Drp1 *in vitro* promotes the transition of T cells to a memory-like phenotype due to the inability of mitochondria to divide (16). The same results were obtained when the glycolysis of T cells was inhibited (36). It should be noted that although knockout of Drp1 appears to promote the conversion of T_E_ to T_M_ by inhibiting glycolysis (37), another study has shown that promoting glycolysis can also increase the formation of memory T cells (38), and the primary metabolism of T_M_ remains controversial (4). Interestingly, the mitochondrial fusion protein Opa1 is not only implicated in effector and memory fate decisions (35), but also affects T cell development in the thymus (39, 40). We believe that both mitochondrial morphologies play a role in T cell development, from maturation to apoptosis, after participating in the immune response. It is crucial to clarify the specific changes in mitochondria and the alteration of pathways during these processes. Whether it is an autoimmune disease that suppresses the immune response, or an anti-tumor treatment that needs to enhance the immune response, more research is needed to clarify the relationship between mitochondrial metabolism and T cell differentiation.

### Drp1-Mediated Mitochondrial Fission and T Cell Death

To avoid autoimmunity, effector T cells die following the immune response. This process is called activation-induced cell death and is regulated by mitochondria. Drp1-mediated mitochondrial fission generates ROS that regulate the transcription and expression of CD95L (FasL), which is essential for CD95 (Fas)-dependent apoptosis (41) (**Figure 1**). In addition, mitochondria also play other roles in apoptosis. The activation of protein kinase A downstream of the T cell receptor (TCR) signal can inhibit autophagy induced by activation-induced cell death, leading to the accumulation and division of damaged mitochondria, thereby releasing cytochrome C and driving apoptosis (42).

## Drp1-Mediated Mitochondrial Division and T Cell Exhaustion in the Tumor Microenvironment (TME)

Because of the lack of T cells or the presence of nonfunctional T cells in the TME, a considerable number of patients show no response to immunotherapy (43). Compared with the secondary lymphoid organs where T cells survive, the TME is always hypoxic. Hypoxic signaling stimulates the generation of markedly heterogeneous blood vessels in the TME (44), which further increases hypoxia due to an uneven lumen (45). In addition, the TME also contains an extracellular matrix and various growth factors (such as hepatocyte growth factor and fibroblast growth factor) secreted by stromal cells and fibroblasts (45, 46). The extracellular matrix can stimulate tumor angiogenesis (47), and growth factors can both promote the growth of malignant cells and act as chemokines to stimulate other cells to migrate to the TME (45). In the TME, T cells undergo continuous TCR stimulation and suffer from nutritional deficiency and hypoxia, leading to a phenotype known as exhaustion (48). Exhausted T cells are characterized by decreased proliferation and functional status, accompanied by increased expression of co-inhibitory molecules (49). Interestingly, exhausted T cells showed damaged mitochondria and abnormal ROS compared with normal T cells (50, 51). This indicates that mitochondrial dynamics are changed during the process of T cell exhaustion, or it may be that these mitochondrial dynamic changes lead to the exhaustion of T cells. Next, we focus on the mitochondrial dynamics of exhausted T cells in the TME and the possible role of Drp1 in these events.

### Persistent Antigen Stimulation Affects T Cell Proliferation and Exhaustion Through Drp1 Changes

**Figure 2:**
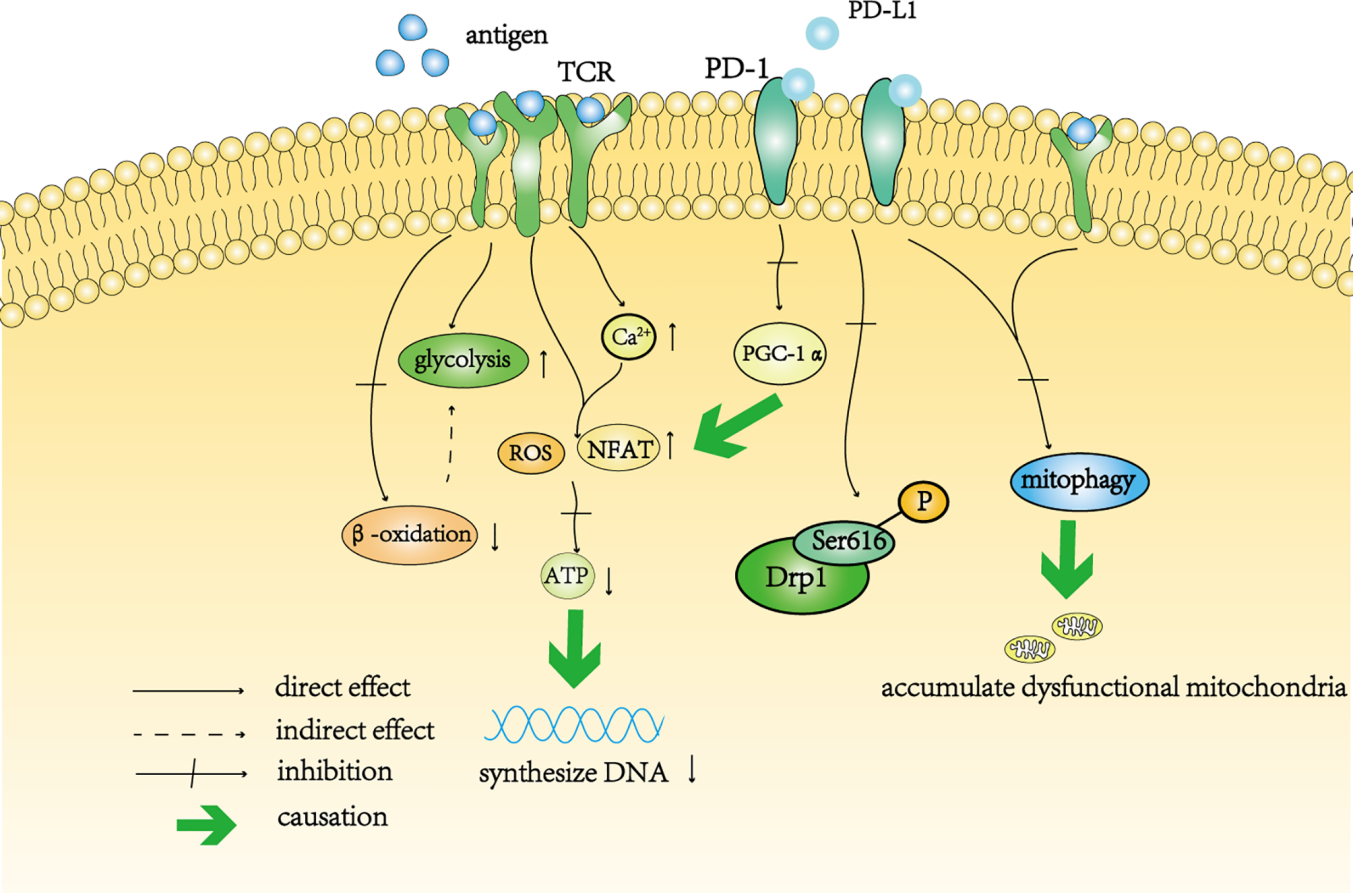
A schematic diagram of T cell depletion caused by continuous antigen stimulation and PD-1. Continuous antigen stimulation increased the level of glycolysis in T cells and inhibited beta-oxidation in these cells. The decrease in oxidative phosphorylation indirectly increases the level of glycolysis. In addition, continuous antigen stimulation not only directly increases the levels of intracellular ROS and NFAT, but also increases the level of intracellular calcium, resulting in the accumulation of intracellular ROS indirectly. High levels of ROS affect the synthesis of ATP, thereby affecting the synthesis of DNA in T cells. Activation of the PD-1 signal pathway inhibits the transcription of PGC-1 α, resulting in an increase in the level of ROS. The PD-1 signal pathway also inhibits the phosphorylation of Drp11 at Ser616. Continuous antigen stimulation cooperates with the PD-1 signaling pathway to prevent mitochondrial autophagy and self-renewal, resulting in the accumulation of dysfunctional mitochondria in T cells.

Some studies have shown that the mitochondrial function of tumor-infiltrating lymphocytes is abnormal (50, 52, 53). Persistent antigen stimulation in the TME leads to the damage of mitochondrial function (**Figure 2**) and further affects the ability of T cells to proliferate (54).

TCR can increase the level of glycolysis by co-stimulation with CD28 receptor (55). *In vitro*, glycolysis increased significantly in T cells after long-term stimulation with antigen (54) (**Figure 2**), which was consistent with transformation of the metabolic mode during the differentiation of effector T cells. However, in this case, the tricarboxylic acid cycle and the synthesis of nucleotide triphosphate were affected, indicating that this continuous stimulation impaired the ability of mitochondrial β-oxidation in T cells (54) (**Figure 2**). The dysfunction of mitochondria leads to the deficiency of ATP synthesis in T cells. It can be speculated that oxidative phosphorylation is impaired in T cells stimulated by antigen, which further aggravates the dependence of T cells on glycolysis (**Figure 2**). In addition, continuous antigen stimulation leads to the accumulation of high levels of ROS in T cells, which directly interferes with ATP synthesis (54) (**Figure 2**). The proliferation of T cells requires the synthesis of DNA and protein, which is inseparable from the production of ATP (56–58). Therefore, although long-term antigen stimulation maintains a high level of glycolysis, the decrease in mitochondrial oxidative phosphorylation affects the ability for T cell proliferation. Long-term stimulation also increases the level of calcium in T cells and further stimulates the increase in mitochondrial ROS (59, 60) and nuclear factor of activated T cells (NFAT) (61, 62), which can directly activate exhaustion-related transcription factors (63) (**Figure 2**). Excessive calcium ions in T cells can also block the interaction between kinesin-1 and microtubules and prevent mitochondria from moving along microtubules in T cells (64, 65). In addition, hypoxia, as another feature of the TME, cooperates with continuous stimulation to impair the respiratory ability of T cells and leads to the expression of co-inhibitory molecules (66). Unfortunately, no research has focused on Drp1 changes in this chronic process.

Interestingly, after long-term stimulation of TCR, mitochondria in T cells exhibited a disrupted membrane structure, decreased mitochondrial cristae length and a decreased number of cristae, which resulted from the accumulation of damaged mitochondria that could not be cleared in a timely manner (67). Damaged mitochondria prevent T cells from being fully utilized and from performing their normal functions (see section *Drp1 and Mitophagy* for more detail). The same result was observed in T cells treated with the Drp1 inhibitor Mdvi-1, that is, mitochondria showed a damaged morphology (67). Previously, we discussed the important role of Drp1-mediated mitochondrial division in T cell energy metabolism and the regulation of ROS production, and emphasized the necessity of mitochondrial relocation in the immune synapse for regulation of the calcium current. Therefore, there is good reason to further explore the changes in Drp1 during this process.

### The Influence of PD-1 on T Cells May Involve Drp1

Programmed cell death protein-1 (PD-1) is a co-inhibitory receptor expressed on the surface of T cells (68), which mediates immunosuppression (69). Upregulation of PD-1 expression is one of the characteristics of exhausted T cells (49). Anti-PD-1 therapy can effectively improve the activity of tumor-infiltrating T cells (70, 71). The effect of PD-1 on tumor-infiltrating T cells is at least partly achieved by inhibiting Drp1.

PD-1 regulates T cell metabolism. First, PD-1 enhances T cell fatty acid β-oxidation and inhibits the T cell glycolysis pathway and amino acid metabolism by changing the length and number of mitochondrial cristae (72, 73). Second, PD-1 inhibits the expression of peroxisome proliferator-activated receptor gamma coactivator 1-alpha (PGC-1α, the most important gene that drives mitochondrial biogenesis), polarizing mitochondria and resulting in the production of high levels of ROS, leading to a metabolic imbalance (74, 75) (**Figure 2**). Metabolic stress not only affects the differentiation of T cells, but also weakens the anti-tumor ability of T cells.

Previous studies have shown that PD-1 can inhibit the proliferation and migration of T cells (76, 77). Simula and colleagues used a mouse tumor model to explore whether PD-1 regulates these processes by affecting Drp1 (78). Their study demonstrated that the PD-1 signal inhibits the division of mitochondria in T cells by inhibiting the phosphorylation of Ser616 sites, and this inhibition is achieved through the ERK and mTOR pathways (76, 78, 79) (**Figure 2**). Compared with thymocyte Drp1-knockout mice, PD-1 inhibitors significantly enhanced the anti-tumor effect of wild-type mice, and this difference was related to the phosphorylation level of Drp1 in T cells (78). Interestingly, consistent with the effect of Drp1 on T cell migration described earlier, Simula and colleagues found that the density of tumor-infiltrating T cells in wild-type mice was higher, which was related to Drp1-mediated polarization and rearrangement of the mitochondria located in the uropod of T cells (78).

These results suggest that in the case of persistent metabolic disorders, PD-1 gradually induces T cells and causes their dysfunction by regulating Drp1. As a key factor of PD-1, Drp1 has great potential as a target for synergistic anti-PD-1 therapy.

### Drp1 and Mitophagy

Regardless of the cause of metabolic stress (i.e., hypoxia, or continuous antigen stimulation and activation of the PD-1 signal pathway), mitochondrial damage ensues. As the main functional organelle in cells, mitochondria are constantly undergoing self-renewal, and damaged mitochondria are removed in a timely manner. Although the proteasome system can degrade mitochondria that undergo reversible damage, the clearance of mitochondria during an immune response mainly occurs through mitophagy (80).

Yu and colleagues showed that antigen stimulation combined with activation of the PD-1 signal impaired the autophagy activity of mitochondria, resulting in the accumulation of a large number of mitochondria in tumor-infiltrating T cells (67). These accumulated mitochondria are characterized by the destruction of the mitochondrial membrane and the structure of the cristae (67). The accumulated mitochondrial mass increases and the membrane potential decreases, indicating that these mitochondria are dysfunctional and depolarized (67). The inability to clear these dysfunctional mitochondria leads to a decrease in mitochondrial adaptation, resulting in the expression of more co-suppressor molecules, such as PD-1, on T cells (67). In addition, further analysis showed that the chromatin accessibility of T cells accumulating a large number of damaged mitochondria changed with DNA methylation, which was related to exhaustion (67).

Drp1-mediated mitochondrial fission plays a role in mitochondrial autophagy (81). Significant inhibition of mitochondrial autophagy was observed in Drp1-negative B cells (82). In addition, the importance of mitochondrial division for mitochondrial autophagy has been observed in a variety of cells (83). It can be speculated that damaged mitochondria are marked and separated by division, and mitochondrial autophagy will target and eliminate depolarized mitochondria (82).

## Therapies Targeting Drp1

In view of the limitations of existing immunotherapy, researchers are exploring alternative targets. Along with an in-depth understanding of metabolic stress in the TME, targeted metabolic anti-tumor therapy has been proven to restore the anti-tumor activity of exhausted T cells (84). Considering the role of Drp1 in the various physiological processes of T cells, the regulation of Drp1 as a target is worth exploring. However, it is precisely because Drp1 is involved in a variety of cellular activities that makes this regulation so complex.

The first challenge encountered in targeting Drp1 is how to regulate its expression. To date, studies have achieved regulation of Drp1 expression by overexpression or knockout of Drp1 at the gene level (85, 86), or by blocking the activation of Drp1 using peptides (87, 88). However, these methods are not suitable for the development of treatments. The regulation of genes may lead to the dysfunction of other cells, and the blocking effect of drugs will affect energy metabolism and the neurological function of the brain (89). The next challenge therefore was to control the level of Drp1 expression. Drp1-mediated mitochondrial fission contributes to the production of effector T cells and enhances the anti-tumor ability of T cells. However, this regulation is not conducive to the production of memory T cells or, therefore, maintaining long-term immune surveillance. Studies have shown that knockdown of Drp1 in tumor cells can significantly inhibit the invasion rate of tumor cells (90–92), but it has so far proven difficult to translate Drp1 targeting of tumor cells into clinical treatments. Advances in nanotechnology have enabled cell-specific drug delivery or direct drug delivery into cells (93), but when the drug acts on cells other than just the tumor cells (i.e., not just immune cells in the TME), it may cause serious side effects. At present, the most promising method is to combine the regulation of Drp1 with adoptive immunotherapy since the regulation of T cells by Drp1 *in vitro* may avoid the various adverse effects *in vivo*.

## Future Directions

Targeting Drp1 of T cells appears to alter T cell function and fate by regulating metabolism, but this process is complex. There appear to be multiple metabolic pathways for both regulatory T cells and memory T cells (4). Therefore, future studies are needed to clarify the changes in signaling pathways during Drp1-mediated mitochondrial fission. However, it is possible to directly manipulate T cell metabolism to meet the needs of antitumor therapy. In addition, chemotherapeutic drugs can target tumor cells in a specific stage of the cell cycle (94). The possibility of modulating mitochondrial dynamics to alter cellular metabolism in such a way as to target tumor cells in a specific stage of the cell cycle, along with the cooperation of chemotherapeutic drugs, is also a direction worth exploring. Radiotherapy can alter the TME and disrupt tumor tissue (95). In this context, modulating Drp1 to improve the function of exhausted T cells in the TME may be synergistic with radiotherapy.

Studies have shown that in MAPK-mutated human tumor cell lines, inhibition of this signaling pathway results in the loss of Drp1, which ultimately leads to excessive mitochondrial fusion (96). Since our goal is to modulate mitochondrial dynamics by enhancing Drp1 expression levels, thereby altering T cell fate and biological behavior, can the same goal be achieved by targeting mitochondrial fusion proteins to inhibit mitochondrial fusion? Studies on breast cancer cells have shown that inhibition of the mitochondrial fusion protein Mfn promotes the accumulation of mitochondria in the lamellipodia region and significantly enhances the migration ability of breast cancer cells, while mitochondrial uncouplers or inhibitors of ATP synthesis can reverse this change (91). However, just as altering Drp1 may trigger changes in multiple signaling pathways, the regulation of mitochondrial fusion proteins also requires caution. Studies have shown that Opa1 has other functions in addition to regulating mitochondrial fusion. Opa1 maintains the integrity of the IMM and cristae, and is also involved in the sequestration of cytochrome c to regulate mitochondrial apoptosis (97). In addition, Opa1 regulates mitochondrial calcium uptake by regulating the coupling between mitochondria and the endoplasmic reticulum (98), which is important for T cell activation. Recently, Liu et al. showed that promoting mitochondrial fusion can improve the function of cardiomyocytes (99), and targeting cell metabolism is becoming a common strategy for the treatment of various diseases. Further research on the regulation of mitochondrial fusion proteins in T cells will undoubtedly confirm the role of mitochondria as key mediators in the regulation of T cells.

## Conclusions

Mitochondria are not only at the center of cell energy metabolism, but they are also responsible for the integration of various signal pathways (3). Immunotherapy is by no means limited to relieving immunosuppression, and reversing the exhaustion of T cells in the TME may overcome the fact that immunotherapy is only effective in some patients. As an important factor in integrating the functions of the mitochondrial network, Drp1 is vital for the correct functioning of T cells. It has been shown that the functional impairment of tumor-infiltrating T cells is related to Drp1, and therefore considering Drp1 as a therapeutic target in the future is a promising strategy. However, it is worth noting that most of the studies so far have simulated the TME *in vitro*, which is very different from the complex environment of tumor patients *in vivo*. In addition, T cells extracted from peripheral blood and tumor-infiltrating T cells have different TCR lineages (100). These factors may affect the translatability of the research into effective therapeutics. Finally, the expression changes and mechanisms of action of Drp1 in exhausted T cells remain to be clarified, and further exploration is needed.

## Author Contributions

JS wrote the manuscript and was the primary author. JM and CH made substantial contributions to designing and revising the article. All authors contributed to the article and approved the submitted version.

## Funding

This study was supported by grants from the 345 Talent Project of Shengjing Hospital and the Liaoning Province Key Research and Development Plan Projects (No. 2020JH2/10300149).

## Conflict of Interest

The authors declare that the research was conducted in the absence of any commercial or financial relationships that could be construed as a potential conflict of interest.

## Publisher’s Note

All claims expressed in this article are solely those of the authors and do not necessarily represent those of their affiliated organizations, or those of the publisher, the editors and the reviewers. Any product that may be evaluated in this article, or claim that may be made by its manufacturer, is not guaranteed or endorsed by the publisher.

## Abbreviations

ATP, adenosine triphosphate; CD95L, ligand of apoptosis factor CD95; Drp1, dynamin-related protein 1; ERK, extracellular signal-regulated kinase; mTOR, mechanistic target of rapamycin; NFAT, nuclear factor of activated T cells; PD-1, programmed cell death protein-1; PGC-1α, peroxisome proliferator-activated receptor gamma coactivator 1-alpha; ROS, reactive oxygen species.

## References

[1] Waldman AD Fritz JM Lenardo MJ. A Guide to Cancer Immunotherapy: From T Cell Basic Science to Clinical Practice. Nat Rev Immunol (2020) 20(11):651–68. doi: 10.1038/s41577-020-0306-5 PMC7238960 32433532

[2] O'Neill LA Kishton RJ Rathmell J. A Guide to Immunometabolism for Immunologists. Nat Rev Immunol (2016) 16(9):553–65. doi: 10.1038/nri.2016.70 PMC5001910 27396447

[3] Rambold AS Pearce EL. Mitochondrial Dynamics at the Interface of Immune Cell Metabolism and Function. Trends Immunol (2018) 39(1):6–18. doi: 10.1016/j.it.2017.08.006 28923365

[4] Raud B Roy DG Divakaruni AS Tarasenko TN Franke R Ma EH. Etomoxir Actions on Regulatory and Memory T Cells Are Independent of Cpt1a-Mediated Fatty Acid Oxidation. Cell Metab (2018) 28(3):504–15.e7. doi: 10.1016/j.cmet.2018.06.002 30043753 PMC6747686

[5] Saravia J Zeng H Dhungana Y Bastardo Blanco D Nguyen TM Chapman NM. Homeostasis and Transitional Activation of Regulatory T Cells Require C-Myc. Sci Adv (2020) 6(1):eaaw6443. doi: 10.1126/sciadv.aaw6443 31911938 PMC6938709

[6] Chen H Detmer SA Ewald AJ Griffin EE Fraser SE Chan DC. Mitofusins Mfn1 and Mfn2 Coordinately Regulate Mitochondrial Fusion and Are Essential for Embryonic Development. J Cell Biol (2003) 160(2):189–200. doi: 10.1083/jcb.200211046 12527753 PMC2172648

[7] Cervantes-Silva MP Cox SL Curtis AM. Alterations in Mitochondrial Morphology as a Key Driver of Immunity and Host Defence. EMBO Rep (2021) 22(9):e53086. doi: 10.15252/embr.202153086 34337844 PMC8447557

[8] Horner SM Liu HM Park HS Briley J Gale M Jr. Mitochondrial-Associated Endoplasmic Reticulum Membranes (MAM) Form Innate Immune Synapses and Are Targeted by Hepatitis C Virus Proc Natl Acad Sci USA (2011) 108(35):14590–5. doi: 10.1073/pnas.1110133108 PMC3167523 21844353

[9] Galloway CA Lee H Yoon Y. Mitochondrial Morphology-Emerging Role in Bioenergetics. Free Radic Biol Med (2012) 53(12):2218–28. doi: 10.1016/j.freeradbiomed.2012.09.035 PMC3594133 23032099

[10] Mishra P Chan DC. Metabolic Regulation of Mitochondrial Dynamics. J Cell Biol (2016) 212(4):379–87. doi: 10.1083/jcb.201511036 PMC4754720 26858267

[11] Labrousse AM Zappaterra MD Rube DA van der Bliek AMC. Elegans Dynamin-Related Protein Drp-1 Controls Severing of the Mitochondrial Outer Membrane. Mol Cell (1999) 4(5):815–26. doi: 10.1016/s1097-2765(00)80391-3 10619028

[12] Yu R Jin SB Lendahl U Nistér M Zhao J. Human Fis1 Regulates Mitochondrial Dynamics Through Inhibition of the Fusion Machinery. EMBO J (2019) 38(8):e99748. doi: 10.15252/embj.201899748 30842096 PMC6463211

[13] Knott AB Perkins G Schwarzenbacher R Bossy-Wetzel E. Mitochondrial Fragmentation in Neurodegeneration. Nat Rev Neurosci (2008) 9(7):505–18. doi: 10.1038/nrn2417 PMC2711514 18568013

[14] Kashatus JA Nascimento A Myers LJ Sher A Byrne FL Hoehn KL. Erk2 Phosphorylation of Drp1 Promotes Mitochondrial Fission and MAPK-Driven Tumor Growth. Mol Cell (2015) 57(3):537–51. doi: 10.1016/j.molcel.2015.01.002 PMC4393013 25658205

[15] Angajala A Lim S Phillips JB Kim JH Yates C You Z. Diverse Roles of Mitochondria in Immune Responses: Novel Insights Into Immuno-Metabolism. Front Immunol (2018) 9:1605. doi: 10.3389/fimmu.2018.01605 30050539 PMC6052888

[16] Simula L Pacella I Colamatteo A Procaccini C Cancila V Bordi M. Drp1 Controls Effective T Cell Immune-Surveillance by Regulating T Cell Migration, Proliferation, and Cmyc-Dependent Metabolic Reprogramming. Cell Rep (2018) 25(11):3059–73.e10. doi: 10.1016/j.celrep.2018.11.018 30540939 PMC6302735

[17] James KD Jenkinson WE Anderson G. T-Cell Egress From the Thymus: Should I Stay or Should I Go? J Leukocyte Biol (2018) 104(2):275–84. doi: 10.1002/jlb.1mr1217-496r PMC6174998 29485734

[18] Moreau HD Piel M Voituriez R Lennon-Duménil AM. Integrating Physical and Molecular Insights on Immune Cell Migration. Trends Immunol (2018) 39(8):632–43. doi: 10.1016/j.it.2018.04.007 29779848

[19] Pandya P Orgaz JL Sanz-Moreno V. Actomyosin Contractility and Collective Migration: May the Force Be With You. Curr Opin Cell Biol (2017) 48:87–96. doi: 10.1016/j.ceb.2017.06.006 28715714 PMC6137077

[20] Campello S Lacalle RA Bettella M Manes S Scorrano L Viola A. Orchestration of Lymphocyte Chemotaxis by Mitochondrial Dynamics. J Exp Med (2006) 203(13):2879–86. doi: 10.1084/jem.20061877 PMC2118173 17145957

[21] Quintana A Schwarz EC Schwindling C Lipp P Kaestner L Hoth M. Sustained Activity of Calcium Release-Activated Calcium Channels Requires Translocation of Mitochondria to the Plasma Membrane. J Biol Chem (2006) 281(52):40302–9. doi: 10.1074/jbc.M607896200 17056596

[22] Quintana A Schwindling C Wenning AS Becherer U Rettig J Schwarz EC. T Cell Activation Requires Mitochondrial Translocation to the Immunological Synapse. Proc Natl Acad Sci USA (2007) 104(36):14418–23. doi: 10.1073/pnas.0703126104 PMC1964825 17726106

[23] Tamás P Hawley SA Clarke RG Mustard KJ Green K Hardie DG. Regulation of the Energy Sensor Amp-Activated Protein Kinase by Antigen Receptor and Ca^2+^ in T Lymphocytes. J Exp Med (2006) 203(7):1665–70. doi: 10.1084/jem.20052469 PMC2118355 16818670

[24] Vaeth M Maus M Klein-Hessling S Freinkman E Yang J Eckstein M. Store-Operated Ca^2+^ Entry Controls Clonal Expansion of T Cells Through Metabolic Reprogramming. Immunity (2017) 47(4):664–79.e6. doi: 10.1016/j.immuni.2017.09.003 29030115 PMC5683398

[25] Blagih J Coulombe F Vincent EE Dupuy F Galicia-Vázquez G Yurchenko E. The Energy Sensor AMPK Regulates T Cell Metabolic Adaptation and Effector Responses in Vivo. Immunity (2015) 42(1):41–54. doi: 10.1016/j.immuni.2014.12.030 25607458

[26] Saravia J Raynor JL Chapman NM Lim SA Chi H. Signaling Networks in Immunometabolism. Cell Res (2020) 30(4):328–42. doi: 10.1038/s41422-020-0301-1 PMC7118125 32203134

[27] Cereghetti GM Stangherlin A Martins de Brito O Chang CR Blackstone C Bernardi P. Dephosphorylation by Calcineurin Regulates Translocation of Drp1 to Mitochondria. Proc Natl Acad Sci USA (2008) 105(41):15803–8. doi: 10.1073/pnas.0808249105 PMC2572940 18838687

[28] Baixauli F Martín-Cófreces NB Morlino G Carrasco YR Calabia-Linares C Veiga E. The Mitochondrial Fission Factor Dynamin-Related Protein 1 Modulates T-Cell Receptor Signalling at the Immune Synapse. EMBO J (2011) 30(7):1238–50. doi: 10.1038/emboj.2011.25 PMC3094108 21326213

[29] Wang R Dillon CP Shi LZ Milasta S Carter R Finkelstein D. The Transcription Factor Myc Controls Metabolic Reprogramming Upon T Lymphocyte Activation. Immunity (2011) 35(6):871–82. doi: 10.1016/j.immuni.2011.09.021 PMC3248798 22195744

[30] Mehta MM Weinberg SE Chandel NS. Mitochondrial Control of Immunity: Beyond ATP. Nat Rev Immunol (2017) 17(10):608–20. doi: 10.1038/nri.2017.66 28669986

[31] Vafai SB Mootha VK. Mitochondrial Disorders as Windows Into an Ancient Organelle. Nature (2012) 491(7424):374–83. doi: 10.1038/nature11707 23151580

[32] Braun MY. The Natural History of T Cell Metabolism. Int J Mol Sci (2021) 22(13):6779. doi: 10.3390/ijms22136779 34202553 PMC8269353

[33] Bailis W Shyer JA Zhao J Canaveras JCG Al Khazal FJ Qu R. Distinct Modes of Mitochondrial Metabolism Uncouple T Cell Differentiation and Function. Nature (2019) 571(7765):403–7. doi: 10.1038/s41586-019-1311-3 PMC6939459 31217581

[34] Xie JH Li YY Jin J. The Essential Functions of Mitochondrial Dynamics in Immune Cells. Cell Mol Immunol (2020) 17(7):712–21. doi: 10.1038/s41423-020-0480-1 PMC7331746 32523116

[35] Buck MD O'Sullivan D Klein Geltink RI Curtis JD Chang CH Sanin DE. Mitochondrial Dynamics Controls T Cell Fate Through Metabolic Programming. Cell (2016) 166(1):63–76. doi: 10.1016/j.cell.2016.05.035 27293185 PMC4974356

[36] Sukumar M Liu J Ji Y Subramanian M Crompton JG Yu Z. Inhibiting Glycolytic Metabolism Enhances CD8+ T Cell Memory and Antitumor Function. J Clin Invest (2013) 123(10):4479–88. doi: 10.1172/jci69589 PMC3784544 24091329

[37] O'Sullivan D van der Windt GJW Huang SC Curtis JD Chang CH Buck MD. Memory CD8(+) T Cells Use Cell-Intrinsic Lipolysis to Support the Metabolic Programming Necessary for Development. Immunity (2018) 49(2):375–6. doi: 10.1016/j.immuni.2018.07.018 PMC6167519 30134202

[38] Phan AT Doedens AL Palazon A Tyrakis PA Cheung KP Johnson RS. Constitutive Glycolytic Metabolism Supports CD8(+) T Cell Effector Memory Differentiation During Viral Infection. Immunity (2016) 45(5):1024–37. doi: 10.1016/j.immuni.2016.10.017 PMC5130099 27836431

[39] Raynor JL Liu C Dhungana Y Guy C Chapman NM Shi H. Hippo/Mst Signaling Coordinates Cellular Quiescence With Terminal Maturation in iNKT Cell Development and Fate Decisions. J Exp Med (2020) 217(6):e20191157. doi: 10.1084/jem.20191157 32289155 PMC7971129

[40] Corrado M Samardžić D Giacomello M Rana N Pearce EL Scorrano L. Deletion of the Mitochondria-Shaping Protein Opa1 During Early Thymocyte Maturation Impacts Mature Memory T Cell Metabolism. Cell Death Differ (2021) 28(7):2194–206. doi: 10.1038/s41418-021-00747-6 PMC8257785 33649469

[41] Röth D Krammer PH Gülow K. Dynamin Related Protein 1-Dependent Mitochondrial Fission Regulates Oxidative Signalling in T Cells. FEBS Lett (2014) 588(9):1749–54. doi: 10.1016/j.febslet.2014.03.029 24681098

[42] Corrado M Mariotti FR Trapani L Taraborrelli L Nazio F Cianfanelli V. Macroautophagy Inhibition Maintains Fragmented Mitochondria to Foster T Cell Receptor-Dependent Apoptosis. EMBO J (2016) 35(16):1793–809. doi: 10.15252/embj.201593727 PMC5010050 27390127

[43] Zhang Y Chen L. Classification of Advanced Human Cancers Based on Tumor Immunity in the Microenvironment (TIME) for Cancer Immunotherapy. JAMA Oncol (2016) 2(11):1403–4. doi: 10.1001/jamaoncol.2016.2450 PMC5492376 27490017

[44] Carmeliet P Jain RK. Molecular Mechanisms and Clinical Applications of Angiogenesis. Nature (2011) 473(7347):298–307. doi: 10.1038/nature10144 21593862 PMC4049445

[45] Hui L Chen Y. Tumor Microenvironment: Sanctuary of the Devil. Cancer Lett (2015) 368(1):7–13. doi: 10.1016/j.canlet.2015.07.039 26276713

[46] Hanahan D Coussens LM. Accessories to the Crime: Functions of Cells Recruited to the Tumor Microenvironment. Cancer Cell (2012) 21(3):309–22. doi: 10.1016/j.ccr.2012.02.022 22439926

[47] Lu P Weaver VM Werb Z. The Extracellular Matrix: A Dynamic Niche in Cancer Progression. J Cell Biol (2012) 196(4):395–406. doi: 10.1083/jcb.201102147 22351925 PMC3283993

[48] Wherry EJ. T Cell Exhaustion. Nat Immunol (2011) 12(6):492–9. doi: 10.1038/ni.2035 21739672

[49] Wherry EJ Kurachi M. Molecular and Cellular Insights Into T Cell Exhaustion. Nat Rev Immunol (2015) 15(8):486–99. doi: 10.1038/nri3862 PMC4889009 26205583

[50] Scharping NE Menk AV Moreci RS Whetstone RD Dadey RE Watkins SC. The Tumor Microenvironment Represses T Cell Mitochondrial Biogenesis to Drive Intratumoral T Cell Metabolic Insufficiency and Dysfunction. Immunity (2016) 45(3):701–3. doi: 10.1016/j.immuni.2016.08.009 27653602

[51] Zhang L Romero P. Metabolic Control of CD8(+) T Cell Fate Decisions and Antitumor Immunity. Trends Mol Med (2018) 24(1):30–48. doi: 10.1016/j.molmed.2017.11.005 29246759

[52] Zhang Y Kurupati R Liu L Zhou XY Zhang G Hudaihed A. Enhancing CD8(+) T Cell Fatty Acid Catabolism Within a Metabolically Challenging Tumor Microenvironment Increases the Efficacy of Melanoma Immunotherapy. Cancer Cell (2017) 32(3):377–91.e9. doi: 10.1016/j.ccell.2017.08.004 28898698 PMC5751418

[53] Fraietta JA Lacey SF Orlando EJ Pruteanu-Malinici I Gohil M Lundh S. Determinants of Response and Resistance to CD19 Chimeric Antigen Receptor (CAR) T Cell Therapy of Chronic Lymphocytic Leukemia. Nat Med (2018) 24(5):563–71. doi: 10.1038/s41591-018-0010-1 PMC6117613 29713085

[54] Vardhana SA Hwee MA Berisa M Wells DK Yost KE King B. Impaired Mitochondrial Oxidative Phosphorylation Limits the Self-Renewal of T Cells Exposed to Persistent Antigen. Nat Immunol (2020) 21(9):1022–33. doi: 10.1038/s41590-020-0725-2 PMC7442749 32661364

[55] Frauwirth KA Riley JL Harris MH Parry RV Rathmell JC Plas DR. The CD28 Signaling Pathway Regulates Glucose Metabolism. Immunity (2002) 16(6):769–77. doi: 10.1016/s1074-7613(02)00323-0 12121659

[56] Sullivan LB Gui DY Hosios AM Bush LN Freinkman E Vander Heiden MG. Supporting Aspartate Biosynthesis Is an Essential Function of Respiration in Proliferating Cells. Cell (2015) 162(3):552–63. doi: 10.1016/j.cell.2015.07.017 PMC4522278 26232225

[57] Bauer DE Hatzivassiliou G Zhao F Andreadis C Thompson CB. ATP Citrate Lyase Is an Important Component of Cell Growth and Transformation. Oncogene (2005) 24(41):6314–22. doi: 10.1038/sj.onc.1208773 16007201

[58] Birsoy K Wang T Chen WW Freinkman E Abu-Remaileh M Sabatini DM. An Essential Role of the Mitochondrial Electron Transport Chain in Cell Proliferation Is to Enable Aspartate Synthesis. Cell (2015) 162(3):540–51. doi: 10.1016/j.cell.2015.07.016 PMC4522279 26232224

[59] Cogliati S Frezza C Soriano ME Varanita T Quintana-Cabrera R Corrado M. Mitochondrial Cristae Shape Determines Respiratory Chain Supercomplexes Assembly and Respiratory Efficiency. Cell (2013) 155(1):160–71. doi: 10.1016/j.cell.2013.08.032 PMC3790458 24055366

[60] Sena LA Li S Jairaman A Prakriya M Ezponda T Hildeman DA. Mitochondria Are Required for Antigen-Specific T Cell Activation Through Reactive Oxygen Species Signaling. Immunity (2013) 38(2):225–36. doi: 10.1016/j.immuni.2012.10.020 PMC3582741 23415911

[61] Martinez GJ Pereira RM Äijö T Kim EY Marangoni F Pipkin ME. The Transcription Factor NFAT Promotes Exhaustion of Activated CD8⁺ T Cells. Immunity (2015) 42(2):265–78. doi: 10.1016/j.immuni.2015.01.006 PMC4346317 25680272

[62] Seo H González-Avalos E Zhang W Ramchandani P Yang C Lio CJ. BATF and IRF4 Cooperate to Counter Exhaustion in Tumor-Infiltrating CAR T Cells. Nat Immunol (2021) 22(8):983–95. doi: 10.1038/s41590-021-00964-8 PMC8319109 34282330

[63] Seo H Chen J González-Avalos E Samaniego-Castruita D Das A Wang YH. TOX and TOX2 Transcription Factors Cooperate With NR4A Transcription Factors to Impose CD8(+) T Cell Exhaustion. Proc Natl Acad Sci USA (2019) 116(25):12410–5. doi: 10.1073/pnas.1905675116 PMC6589758 31152140

[64] Quintana A Hoth M. Mitochondrial Dynamics and Their Impact on T Cell Function. Cell calcium (2012) 52(1):57–63. doi: 10.1016/j.ceca.2012.02.005 22425631

[65] Wang X Schwarz TL. The Mechanism of Ca2+ -Dependent Regulation of Kinesin-Mediated Mitochondrial Motility. Cell (2009) 136(1):163–74. doi: 10.1016/j.cell.2008.11.046 PMC2768392 19135897

[66] Scharping NE Rivadeneira DB Menk AV Vignali PDA Ford BR Rittenhouse NL. Mitochondrial Stress Induced by Continuous Stimulation Under Hypoxia Rapidly Drives T Cell Exhaustion. Nat Immunol (2021) 22(2):205–15. doi: 10.1038/s41590-020-00834-9 PMC7971090 33398183

[67] Yu YR Imrichova H Wang H Chao T Xiao Z Gao M. Disturbed Mitochondrial Dynamics in CD8(+) Tils Reinforce T Cell Exhaustion. Nat Immunol (2020) 21(12):1540–51. doi: 10.1038/s41590-020-0793-3 33020660

[68] Xia Y Jeffrey Medeiros L Young KH. Signaling Pathway and Dysregulation of PD1 and Its Ligands in Lymphoid Malignancies. Biochim Biophys Acta (2016) 1865(1):58–71. doi: 10.1016/j.bbcan.2015.09.002 26432723 PMC4733614

[69] Sharpe AH Pauken KE. The Diverse Functions of the PD1 Inhibitory Pathway. Nat Rev Immunol (2018) 18(3):153–67. doi: 10.1038/nri.2017.108 28990585

[70] Sun C Mezzadra R Schumacher TN. Regulation and Function of the PD-L1 Checkpoint. Immunity (2018) 48(3):434–52. doi: 10.1016/j.immuni.2018.03.014 PMC7116507 29562194

[71] Iwai Y Hamanishi J Chamoto K Honjo T. Cancer Immunotherapies Targeting the PD-1 Signaling Pathway. J Biomed Sci (2017) 24(1):26. doi: 10.1186/s12929-017-0329-9 28376884 PMC5381059

[72] Patsoukis N Bardhan K Chatterjee P Sari D Liu B Bell LN. PD-1 Alters T-Cell Metabolic Reprogramming by Inhibiting Glycolysis and Promoting Lipolysis and Fatty Acid Oxidation. Nat Commun (2015) 6:6692. doi: 10.1038/ncomms7692 25809635 PMC4389235

[73] Ogando J Sáez ME Santos J Nuevo-Tapioles C Gut M Esteve-Codina A. PD-1 Signaling Affects Cristae Morphology and Leads to Mitochondrial Dysfunction in Human CD8(+) T Lymphocytes. J Immunother Cancer (2019) 7(1):151. doi: 10.1186/s40425-019-0628-7 31196176 PMC6567413

[74] Scharping NE Menk AV Moreci RS Whetstone RD Dadey RE Watkins SC. The Tumor Microenvironment Represses T Cell Mitochondrial Biogenesis to Drive Intratumoral T Cell Metabolic Insufficiency and Dysfunction. Immunity (2016) 45(2):374–88. doi: 10.1016/j.immuni.2016.07.009 PMC5207350 27496732

[75] Bengsch B Johnson AL Kurachi M Odorizzi PM Pauken KE Attanasio J. Bioenergetic Insufficiencies Due to Metabolic Alterations Regulated by the Inhibitory Receptor Pd-1 Are an Early Driver of CD8(+) T Cell Exhaustion. Immunity (2016) 45(2):358–73. doi: 10.1016/j.immuni.2016.07.008 PMC4988919 27496729

[76] Patsoukis N Brown J Petkova V Liu F Li L Boussiotis VA. Selective Effects of PD-1 on Akt and Ras Pathways Regulate Molecular Components of the Cell Cycle and Inhibit T Cell Proliferation. Sci Signal (2012) 5(230):ra46. doi: 10.1126/scisignal.2002796 22740686 PMC5498435

[77] Zinselmeyer BH Heydari S Sacristán C Nayak D Cammer M Herz J. PD-1 Promotes Immune Exhaustion by Inducing Antiviral T Cell Motility Paralysis. J Exp Med (2013) 210(4):757–74. doi: 10.1084/jem.20121416 PMC3620347 23530125

[78] Simula L Antonucci Y Scarpelli G Cancila V Colamatteo A Manni S. PD-1-Induced T Cell Exhaustion Is Controlled by a Drp1-Dependent Mechanism. Mol Oncol (2022) 16(1):188–205. doi: 10.1002/1878-0261.13103 34535949 PMC8732338

[79] Parry RV Chemnitz JM Frauwirth KA Lanfranco AR Braunstein I Kobayashi SV. CTLA-4 and PD-1 Receptors Inhibit T-Cell Activation by Distinct Mechanisms. Mol Cell Biol (2005) 25(21):9543–53. doi: 10.1128/mcb.25.21.9543-9553.2005 PMC1265804 16227604

[80] Xu Y Shen J Ran Z. Emerging Views of Mitophagy in Immunity and Autoimmune Diseases. Autophagy (2020) 16(1):3–17. doi: 10.1080/15548627.2019.1603547 30951392 PMC6984455

[81] Kanki T Wang K Baba M Bartholomew CR Lynch-Day MA Du Z. A Genomic Screen for Yeast Mutants Defective in Selective Mitochondria Autophagy. Mol Biol Cell (2009) 20(22):4730–8. doi: 10.1091/mbc.e09-03-0225 PMC2777103 19793921

[82] Twig G Elorza A Molina AJ Mohamed H Wikstrom JD Walzer G. Fission and Selective Fusion Govern Mitochondrial Segregation and Elimination by Autophagy. EMBO J (2008) 27(2):433–46. doi: 10.1038/sj.emboj.7601963 PMC2234339 18200046

[83] Tong M Zablocki D Sadoshima J. The Role of Drp1 in Mitophagy and Cell Death in the Heart. J Mol Cell Cardiol (2020) 142:138–45. doi: 10.1016/j.yjmcc.2020.04.015 PMC7545744 32302592

[84] Guo Y Xie YQ Gao M Zhao Y Franco F Wenes M. Metabolic Reprogramming of Terminally Exhausted CD8(+) T Cells by IL-10 Enhances Anti-Tumor Immunity. Nat Immunol (2021) 22(6):746–56. doi: 10.1038/s41590-021-00940-2 PMC7610876 34031618

[85] Frank S Gaume B Bergmann-Leitner ES Leitner WW Robert EG Catez F. The Role of Dynamin-Related Protein 1, a Mediator of Mitochondrial Fission, in Apoptosis. Dev Cell (2001) 1(4):515–25. doi: 10.1016/s1534-5807(01)00055-7 11703942

[86] Barsoum MJ Yuan H Gerencser AA Liot G Kushnareva Y Gräber S. Nitric Oxide-Induced Mitochondrial Fission Is Regulated by Dynamin-Related Gtpases in Neurons. EMBO J (2006) 25(16):3900–11. doi: 10.1038/sj.emboj.7601253 PMC1553198 16874299

[87] Qi X Qvit N Su YC Mochly-Rosen D. A Novel Drp1 Inhibitor Diminishes Aberrant Mitochondrial Fission and Neurotoxicity. J Cell Sci (2013) 126(Pt 3):789–802. doi: 10.1242/jcs.114439 23239023 PMC3619809

[88] Cassidy-Stone A Chipuk JE Ingerman E Song C Yoo C Kuwana T. Chemical Inhibition of the Mitochondrial Division Dynamin Reveals Its Role in Bax/Bak-Dependent Mitochondrial Outer Membrane Permeabilization. Dev Cell (2008) 14(2):193–204. doi: 10.1016/j.devcel.2007.11.019 18267088 PMC2267902

[89] Kornfeld OS Qvit N Haileselassie B Shamloo M Bernardi P Mochly-Rosen D. Interaction of Mitochondrial Fission Factor With Dynamin Related Protein 1 Governs Physiological Mitochondrial Function in Vivo. Sci Rep (2018) 8(1):14034. doi: 10.1038/s41598-018-32228-1 30232469 PMC6145916

[90] Ma JT Zhang XY Cao R Sun L Jing W Zhao JZ. Effects of Dynamin-Related Protein 1 Regulated Mitochondrial Dynamic Changes on Invasion and Metastasis of Lung Cancer Cells. J Cancer (2019) 10(17):4045–53. doi: 10.7150/jca.29756 PMC6692611 31417649

[91] Zhao J Zhang J Yu M Xie Y Huang Y Wolff DW. Mitochondrial Dynamics Regulates Migration and Invasion of Breast Cancer Cells. Oncogene (2013) 32(40):4814–24. doi: 10.1038/onc.2012.494 PMC3911914 23128392

[92] Xie Q Wu Q Horbinski CM Flavahan WA Yang K Zhou W. Mitochondrial Control by Drp1 in Brain Tumor Initiating Cells. Nat Neurosci (2015) 18(4):501–10. doi: 10.1038/nn.3960 PMC4376639 25730670

[93] Farokhzad OC Langer R. Impact of Nanotechnology on Drug Delivery. ACS nano (2009) 3(1):16–20. doi: 10.1021/nn900002m 19206243

[94] Sun Y Liu Y Ma X Hu H. The Influence of Cell Cycle Regulation on Chemotherapy. Int J Mol Sci (2021) 22(13):6923. doi: 10.3390/ijms22136923 34203270 PMC8267727

[95] Jarosz-Biej M Smolarczyk R Cichoń T Kułach N. Tumor Microenvironment as a “Game Changer” in Cancer Radiotherapy. Int J Mol Sci (2019) 20(13):3212. doi: 10.3390/ijms20133212 PMC6650939 31261963

[96] Serasinghe MN Wieder SY Renault TT Elkholi R Asciolla JJ Yao JL. Mitochondrial Division Is Requisite to Ras-Induced Transformation and Targeted by Oncogenic MAPK Pathway Inhibitors. Mol Cell (2015) 57(3):521–36. doi: 10.1016/j.molcel.2015.01.003 PMC4320323 25658204

[97] Olichon A Baricault L Gas N Guillou E Valette A Belenguer P. Loss of OPA1 Perturbates the Mitochondrial Inner Membrane Structure and Integrity, Leading to Cytochrome C Release and Apoptosis. J Biol Chem (2003) 278(10):7743–6. doi: 10.1074/jbc.C200677200 12509422

[98] Cartes-Saavedra B Macuada J Lagos D Arancibia D Andrés ME Yu-Wai-Man P. OPA1 Modulates Mitochondrial Ca^2+^ Uptake Through Er-Mitochondria Coupling. Front Cell Dev Biol (2021) 9:774108. doi: 10.3389/fcell.2021.774108 35047497 PMC8762365

[99] Liu C Han Y Gu X Li M Du Y Feng N. Paeonol Promotes Opa1-Mediated Mitochondrial Fusion *Via* Activating the CK2α-Stat3 Pathway in Diabetic Cardiomyopathy. Redox Biol (2021) 46:102098. doi: 10.1016/j.redox.2021.102098 34418601 PMC8385203

[100] Li H van der Leun AM Yofe I Lubling Y Gelbard-Solodkin D van Akkooi ACJ. Dysfunctional CD8 T Cells Form a Proliferative, Dynamically Regulated Compartment Within Human Melanoma. Cell (2019) 176(4):775–89.e18. doi: 10.1016/j.cell.2018.11.043 30595452 PMC7253294

